# Effects of a Psychosocial Intervention on the Subjective Experiences of Children Living with Atopic Dermatitis: A Qualitative Study in Hong Kong †

**DOI:** 10.3390/children10020395

**Published:** 2023-02-17

**Authors:** Qian-Wen Xie

**Affiliations:** 1 Department of Social Welfare and Risk Management, School of Public Affairs, Zhejiang University, Hangzhou 310058, China; xieqianwen377@163.com; 2 Research Center for Common Prosperity, Future Regional Development Laboratory, Innovation Center of Yangtze River Delta, Zhejiang University, Jiaxing 314100, China; 3 The Center of Social Welfare and Governance, Zhejiang University, Hangzhou 310058, China

**Keywords:** children, atopic dermatitis, psychosocial intervention, qualitative research, child-centered inquiry, Hong Kong

## Abstract

Nonpharmaceutical interventions are important for addressing the psychosocial needs of children living with atopic dermatitis (AD). The current study aimed to investigate the effects of an integrative body–mind–spirit (IBMS) intervention on the subjective experiences of affected children and explore the mechanisms underlying the effects. Using a drawing-based, qualitative approach, the current study conducted two rounds of interviews with 13 children (aged 8–12 years) diagnosed with moderate or severe AD before and after they attended the IBMS intervention. Data were analyzed by using the thematic analysis method. The IBMS intervention worked by changing participants’ perceptions at the cognitive level, improving their coping strategies at the behavioral level, and constructing their social support network at the environmental level. Cognitive, behavioral, and environmental factors might mediate the relationships between the IBMS intervention and participants’ psychological and physical outcomes. This study highlighted the wider inclusion of child-centered qualitative research in the evaluation of the effects of psychosocial interventions designed for children.

## 1. Introduction

Atopic dermatitis (AD) affects 15–30% of children and adolescents worldwide [1]. The constant and intense pruritus, disfigurement, and chronic and relapsing nature of AD impose a significant psychosocial burden on affected children [2,3]. Nonpharmaceutical interventions targeting psychological and social functioning are essentially important for this group of children as pharmaceutical treatments targeted at physical symptoms of the disease may be insufficient for addressing their needs [4]. However, evidence of the effects of nonpharmaceutical interventions for children living with AD is very scarce.

A disconnection between children and services provided to them might exist as the views of children have rarely been addressed in previous evaluations of the interventions designed for them. Although quantitative studies, especially randomized controlled trials (RCTs), could clearly detect the causal relationship between the interventions and predefined outcomes of children’s health, how children themselves perceived the effects of the interventions was unknown. Children’s outcomes have been most commonly rated by adults, particularly their parents or caregivers, rather than children themselves when measuring intervention effects in previous studies [5]. Even when using self-reported scales, preselected standardized scales might still limit children’s reporting of their subjective perceptions of interventions [6]. Quantitative evaluations might yield limited insights into how these interventions work or do not work [7].

According to the United Nations Conventions on the Rights of the Child, children have rights to have their voices heard and to actively participate in all matters that are relevant for them [8]. Given the growth of the participatory rights perspective and the “new childhood studies”, much contemporary research has emphasized seeking information directly from children rather than seeking information about them [9,10]. To appreciate the perspectives of children receiving services and to respect their uniqueness, bringing their voices into evaluation studies through child-appropriate approaches is likely to be of greater importance [11,12].

### The Present Study

The current study aimed to listen to the views of children living with AD and investigate the impact of a psychosocial intervention on their subjective experiences. Based on the integrative body–mind–spirit (IBMS) model, a research team at the University of Hong Kong designed a psychosocial intervention for children living with AD and their parents in Hong Kong. The IBMS model emphasizes the balance among an individual’s physical health, emotion, spirituality, and large environmental systems [13]. Using a parallel group format, the IBMS intervention provided children living with AD and their parents with six-session workshops (one session per week and 3 h per session). Children’s sessions focused on self-identity and self-appreciation (session 1); disease identity (session 2); recognition and expression of emotion (session 3); resilience capacity (session 4); personal strengths (session 5); and support networks (session 6). Each session included a thirty-minute period for parent–child joint activities. Professional social workers provided participants the intervention at nine social service centers of two nongovernmental organizations (NGOs).

## 2. Materials and Methods

### 2.1. Study Design

The current study employed a drawing-based, qualitative approach. Qualitative methodologies have been widely used to explore people’s subjective views and experiences in real-life situations [14]. Qualitative research has been employed increasingly alongside trials of interventions due to the need to probe the mechanisms behind the effects [15]. As one of the participatory methodological approaches, the drawing-based approach has been perceived as sensitive to the discussion of children’s perceptions and have the power to depict their representational world [16].

### 2.2. Participants

Participants were recruited from the 55 children (aged 6–12 years) who registered and planned to participate in the IBMS intervention. Maximum variation sampling was employed to capture a wide range of perceptions of children. Table 1 presents the sampling process. Participants were eligible to participate in the present qualitative study if they were aged 8 to 12 years and attended the IBMS workshops. According to children’s major stages of artistic development, children over the age of 7 are able to make schematic drawings [17]. Thirty-five eligible children were divided into 20 categories based on their age (8, 9, 10, 11, and 12 years), gender (male vs. female), and severity of AD (moderate vs. severe). The severity of AD was measured by the SCORing Atopic Dermatitis (SCORAD) Index (maximum total score = 103) [18]. Moderate AD was defined as a score greater than 25 and less than 50, and severe AD was defined as a score greater than 50. When there was more than one child in one category, one child was selected randomly as a potential participant. Thus, 16 children were selected as potential participants. Sampling was conducted at both the individual and service center levels. Since no child was selected from service center A after sampling at the individual level, one child (A12) was randomly selected from three eligible children (A07, A11, and A12) who received the IBMS intervention in service center A. The 17 children and their parents were called and asked if they would like to participate in this qualitative study. H14 refused the interview. H09 was recruited because his characteristics were mostly similar to those of H14. After the first round of interviews, four boys (B05, D02, H09 and K12) did not participate in the IBMS intervention due to time conflicts. The final sample of this qualitative study was 13 children living with AD.

The background characteristics of the 13 participants (eight girls and five boys) are provided in Table 2. Nine participants had severe AD, and the remaining four had moderate AD. Nine participants were diagnosed with AD before being one year old. All participants received various types of treatments, such as ointment, partial medication, wet wrap therapy, oral antibiotics, and traditional Chinese medicine (TCM). Four participants had other health conditions, such as allergic rhinitis (H10), asthma (G16), ADHD (C15), and ASD (D08). The participants were from diverse family backgrounds. One participant (G12) was from a low-income family with a monthly household income less than HKD 20,000. Most families had more than one child. Six families had more than one child diagnosed with AD.

### 2.3. Data Collection

Two rounds of semi-structured interviews combined with a draw-and-explain technique were employed to collect data [2]. The first round of interviews was conducted from September to October 2017 before the IBMS intervention, and the second round was conducted between November 2017 and February 2018 after the intervention. In each interview, a child was provided with a set of 12 colored pencils and asked to complete a drawing task named “drawing your AD”. In this task, a child was asked to draw a picture about whatever he or she would like to draw when thinking of his or her AD on an A4 sheet of white paper. After the task, a child was asked to verbally explain his or her drawing. A set of open-ended questions was also asked during the process, such as “Could you please tell me more about this drawing?” and “How do you feel about this?” Questions on the experiences or perceptions regarding the IBMS intervention were asked by the end of the second-round interviews. The interview protocol and procedures were refined through two pilot interviews. Five investigators with professional background in social work or psychology received training provided by the author. They conducted the individual interviews with children in a distraction-free room at each service center under the supervision of the author. All interviews were audio-recorded. The tape-recorded interviews lasted from 22 to 88 min (mean duration: 53 min). The interviews were conducted in Chinese and transcribed verbatim.

### 2.4. Data Analysis

Transcripts were coded using NVivo v12. The two rounds of data were separately analyzed by using the thematic analysis method [19]. After obtaining a sense of the database, significant statements were grouped into codes, which were then combined into themes. An a priori codebook was developed by the author through this inductive process. A research assistant independently analyzed the first round of data by applying this codebook to reduce bias. Disagreements were resolved through discussions leading to a revised codebook. Then, the author deductively analyzed the second data set according to the codebook. In addition, new meaning units and themes regarding children’s experiences or perceptions of the IBMS interventions emerged, leading to a finalized codebook (see Table 3), that included main themes such as “physical experiences”, “psychological experiences”, “cognitive experiences”, “social experiences”, “coping strategies”, and “experiences in IBMS”. To determine the changes in participants’ lived experiences before and after they participated in the IBMS intervention, we compared the overall themes and meaning units, and the themes and codes in each individual’s interviews across two time points. Changes in participants’ subjective experiences over time were described to explore the impact of the intervention. Although visual artworks were not analyzed formally, they helped to understand the content and context of participants’ verbal expressions.

## 3. Results

This section describes the changes in participants’ subjective experiences at cognitive level (i.e., perceptions), behavioral level (i.e., coping strategies), environmental level (i.e., interpersonal relationships), and outcome levels (i.e., psychological and physical experiences) over time before and after attending the IBMS intervention.

### 3.1. Changes at the Cognitive Level

*Changes in the perceptions of AD.* The image of AD was exclusively bad in the perspectives of almost all participants in the first interviews; while six of them reconstructed the meaning of AD and developed new, relatively positive understandings of AD after the IBMS intervention (e.g., C14, C15, D06, E02, G12, and H10). For example, D06 perceived AD as an exclusively “bad person” in the first interview; however, she perceived it as “an ordinary person” in the second interview. In the first interview with C14, she perceived AD as a devil who stole the key to the lock of her interpersonal relationships since she experienced terrible peer relationships in school and the discrimination in the community. Comparatively, she perceived AD as having two sides, showing higher acceptance of her skin disease. She explained her second drawing:

This (the pink one on the left side) is very itchy. This (the red one on the right side) is comfortable, not itchy. One is on the left side and one is on the right side… because AD is just one thing. It is the same thing but there are two sides… The pink one represents mildness and gentleness. The color is not that heavy… The red one represents bleeding, itchy, and sensitivity… They are dinosaurs because they are fierce. It (AD) is very fierce, but it has a gentle side… I am inseparable from both of them… I’m standing in the middle. It (AD) is both, an enemy and a friend… (C14, female, 12 years old, severe)

Many participants perceived living with AD as “very miserable” in the first interviews, while two of them reinterpreted the role of AD in their lives after the IBMS intervention (e.g., E02 and G12). For example, G12 focused on only challenges and restrictions caused by AD in the first interview by drawing how AD greatly impacted his daily life, sports, and relationships with others. It seemed that he was overshadowed by his skin disease, and there was no way out. In the second interview, however, his spirit seemed to be transformed from restrictions to freedom. He drew Doraemon’s anywhere door and bamboo copter, which could help him break the shackles created by his skin disease. Additionally, he preferred the things he liked such as his hobbies instead of AD as his core memory. He used the film *Inside Out* to explain the role of AD in his current life:

AD is not a core memory… It (AD) can be taken away…Core memory is a good thing… The core memory is an island. When I started playing football, I really loved it. The island is based on my preference. I like playing football, and then I have the football island… At the beginning, there are memories of AD, but as you grow up, only the core memory stays here… Some things can be forgotten. (G12, male, 11 years old, severe)

*Changes in the perceptions of AD treatments.* In the first interviews, most participants perceived the various frequent treatments as unbearable. After the IBMS intervention, however, three participants began to find benefits from treatments that they used to deeply hate (e.g., A12, D08, and G16). For example, in the first interview, G16 said that he hated the bitter Chinese herbal tea the most and perceived drinking Chinese medicine as the hardest challenge in his experiences of living with AD. However, he showed positive attitudes and described the benefits of Chinese herbal tea on his skin condition in the second one. Rubbing ointments was an issue that made most participants feel highly stressed out. Some of them complained of the “sticky” and “uncomfortable” feelings of applying ointments in the first interviews (e.g., A12, E02, H08, and H20). Comparatively, three participants displayed different opinions in the second interviews (e.g., A12, D08, and E02). For example, E02 found additional benefits from applying ointments as she could spend more time chatting with my mother when rubbing ointments at night. Additionally, A12 recognized it as a necessary effort to gain better skin conditions:

I think it (AD) won’t be cured, but it may be better… I really want it to be better. In addition to speaking, I must work hard… rubbing the ointments on and not being lazy. (A12, female, 9 years old, severe)

*Changes in the perceptions of self.* In the first interviews, participants usually presented an other-centered self as they paid considerable attention to other people’s comments and judgments. As a result, participants expressed self-hate of their skin and perceived it as “dirty” and “disgusting” (e.g., E02 and K07). Participants also tended to deny themselves, identified themselves as being incapable and powerless, and saw themselves as victims of AD (e.g., A12, C14, C15, H08, and K07). Two even thought that it was reasonable for classmates and teachers to dislike them because they had AD (e.g., H08 and B05). After the IBMS intervention, however, some participants showed more self-awareness and developed a larger sense of “self”. For example, A12 drew a person beside her who usually judged her skin in her first drawing while she did not draw this person in her second drawing. According to her, comments of other people that used to hurt her deeply seemed not to be that important. She paid more attention to herself and her own feelings in the second interview. Similarly, C14 wished that she could be an “ordinary person” or even a “foolish person” rather than a person with AD in the first interview. While in the second one, she said “I am I, even though I have AD.” A more positive self-concept might have resulted from positive interactions with other children in the IMBS group. For example, E02 described how her kindness to other children made her satisfied with herself:

I learned to play with other people and learned social skills… Sometimes we went to choose some prizes, and a child wanted that color of the prize, I also wanted that color… I often give these to others… I feel very happy… because I make other people happy. I am very satisfied with myself. (E02, female, 10 years old, moderate)

### 3.2. Changes at the Behavioral Level

*Increased strategies in coping with itching and scratching.* Severe itching caused by AD provoked the desire to scratch, subsequently leading to an itch–scratch cycle. Two participants indicated that scratching was like “turning on a light” that could not be stopped even when they realized that they were injuring themselves (e.g., E02 and C14). While four participants indicated that they learned strategies for handling scratching, such as distracting one’s attention, from the IBMS workshops (e.g., A12, C15, D06, and H10). As A12 stated, “I may learn more about how to distract myself. Don’t focus on AD, and don’t scratch it all day”. H20 drew “itching” as the core theme in two interviews, but her responses to itching changed. In the first interview, she said that she could not stop scratching, and then she could not sleep. In the second interview, she proudly shared her creative strategy that she learned from other children in the group to prevent herself from scratching: “I pressed my hands with my buttocks because I use hands to scratch. Thus, I don’t let my hands scratch it”. Because of the new strategy, she could finally focus on what the teacher said in the class rather than her itchy skin.

*Increased strategies in coping with emotional stress.* In the first interviews, most participants complained about intense and constant pruritus, which played an important role in determining their mood and emotions, causing irritability and stress. Three participants showed improved competence with emotion regulation compared with the first interviews (e.g., B06, D08, and H10). They indicated that they learned self-relaxation and calming through techniques such as mindfulness breathing in the IBMS group. As H10 said, “I learned how to calm down when I feel stressed, and something related to emotions”. Participants could also feel the effect of their relaxed emotions on their skin.

I learned to relax myself. My mood is relaxed. I take a deep breath. I feel comfortable. My AD seems to be weak. (D08, male, 9 years old, moderate)

*Increased strategies for coping with bullying and discrimination.* In the first interviews, almost all participants indicated that they suffered problematic relationships with peers as they experienced frequent verbal, social, and even physical bullying in their schools and discrimination and stigmatization in their neighborhoods. Participants felt helpless and overwhelmed due to deteriorated interpersonal relationships. After the IBMS intervention, nine participants found new meaning of interpersonal relationships (e.g., A12, B05, C14, D06, E02, G16, H09, H20, and K12). For example, D06 focused on her suffering of being isolated and discriminated against by classmates in the first interview while she developed new insights into the actions of people who bullied her in the second interview. As she said in the second interview:

I think they are very mediocre. Sometimes people collectively say bad things about other people. Some people themselves are good, but they all join in. Being in a group (to bully) may be just for fun. (D06, female, 8 years old, severe)

Similarly, compared to the first interview, E02′s hatred disappeared as she developed a new understanding of true friendship and could forgive those classmates who discriminated and bullied her. E02 described her feelings in the second interview:

I feel that normal people will not look at your skin or care if you have AD…If you have AD, don’t cover it. If you think that a person is really a friend, you can tell her directly that you have AD. Normally, if the person is a true and good friend, he or she won’t be particularly concerned about your appearance… Maybe these students (who bullied me) were too young at that time. They didn’t understand, and they couldn’t understand. This was also normal. I feel OK now. I am not angry now… It is actually a very small thing. (E02, female, 10 years old, moderate)

Four participants preferred to face comments or discrimination with a mindful attitude (e.g., C14, D06, E02, and H10). When classmates called C14 a grandmother because of her skin and face, she responded that, “I didn’t feel anything. Let him say it… I’d let it go. It doesn’t matter”. H10 also indicated that he learnt how not to have to fight with others when someone laughs at his skin. Some others preferred to explain their disease to others to gain more understanding when facing their questions or comments. For example, K07 was struggling in the first interview due to not having friend at school, while in the second interview, she said that she would explain her disease to classmates and let them know that AD was not infectious. Some preferred to directly revolt against difficulties. Additionally, B06 drew and described his vulnerability and helplessness in facing verbal and physical bullying at school due to his skin condition in the first interview. In the second interview, he described how he finally solved the problem of verbal bullying by his classmates:

I left this annoying situation this month… I reported them for sending spam in the (WhatsApp) group… I destroyed this group… They verbally bullied me… I reported them and waited until the administrator removed this group. I feel very happy. (B06, male, 12 years old, moderate)

### 3.3. Changes at the Environmental Level

*Changes in parent–child interactions.* Many participants focused on conflicts with parents about AD treatments in the first interviews as most parents tended to give instructions instead of working together with them in cocreation (e.g., A12, C15, E02, H08, H20, G16, and H09). Five participants said that their parents even criticized, blamed, scolded, and beat them when they were scratching (e.g., D06, E02, H08, G12, and G16). Conflicts with parents on treatments made children feel even more angry, distressed, and annoyed. In comparison, they obviously preferred to describe the positive interactions with parents during the second interviews. Most participants expressed that they enjoyed joint activities with parents in the IBMS intervention, including mindful jar making, problem-solving games, mutual gift presentation, and love/appreciation dialog. Three participants thought that making gifts together was the happiest activity, and gifts can be given to their own parents (e.g., A12, D06, and G16). As G16 described:

I made the handicraft with dad. He helped me to make it, and we showed it in front of everyone in the class. It’s a gift that I prepared for dad. We went back home and mounted the gift. I feel very happy and proud. (G16, male, 8 years old, severe)

Three participants also indicated that they received more support and understanding from parents after attending the IBMS intervention (e.g., B06, C15, and H08). Due to the increased trust, three participants began to proactively communicate with parents about the difficulties they experienced at school, leading to more relaxed psychological and emotional status (e.g., A12, B06, and E02). As B06 indicated:

They care more about me… (They did) less in the past. Now they care more than before. They are now often concerned about my condition in the school… If you didn’t talk about it (issues that happen at school) before, you always felt tired. I don’t know why. Now I’m not as tired after I say it. I don’t feel (as) tired as before. (B06, male, 12 years old, moderate)

*Changes in peer relationships.* In the first interviews, almost all participants indicated that they suffered problematic relationships with peers. Most participants indicated that they had no friends at school and felt very lonely. Their classmates “completely ignored” them, and “avoided”, “dodged”, “stepped aside”, or “went away” when they saw them, “just like seeing a ghost” (e.g., C14, D06, E02, H08, B05, C15, D08, and G12). They perceived being rejected and isolated by peers as a pervasive problem affecting them. Fortunately, they could play together with other children as a team in the IBMS group. By the end of the second-round interviews, when asking questions on the experiences or perceptions regarding the IBMS intervention, ten of 13 participants perceived playing as the most impressive component of the intervention (e.g., B06, C14, C15, D06, E02, G12, G16, H08, H20, and K07). They vividly recalled and described various specific games they played in the IBMS group. Nine participants said that they made new friends in the group in the second interview (e.g., A12, B06, D06, D08, E02, G16, H08, H20, and K07). Being accepted by fellow members with the same skin conditions in the group produced feelings of warm and nurturing support for participants. For example, B06 indicated that he was very lonely in the first interview while he expressed his greater appreciation for his life as he could have new friends in the IBMS group. As he described, “My drawing is colorful, so my life is very colorful”. K07 and A12 also described their experiences of having new friends:

All of them are friends… It It’s easy to get along with them… I felt very comfortable when chatting with them… We talked about a lot of things… I can chat with them very comfortably; there is not so much pressure… Everyone has AD, and there is not so much pressure when being together with them. (K07, female, 11 years old, severe)

We are friends… Maybe if they already have it (AD), they won’t mind others… I feel very happy. (A12, female, 9 years old, severe)

### 3.4. Changes at the Outcome Level

*Changes in psychological experiences.* In the first interviews, most participants showed great psychological disturbances caused by their intense itchiness, sleep disturbances, restrictions in daily life, school bullying, and discrimination in the community. Their predominant emotions were anger, annoyance, sadness, worry, fear, embarrassment, loneliness, and confusion. In the second interviews, many participants expressed much more positive emotions. They expressed their happiness and enjoyment of the group activities. As D06 said:

I learned teamwork. We did some exercises and did that together. I feel happy… not only myself… working with other people (with the same illness) and not alone. (D06, female, 8 years old, severe)

Besides the positive emotions, four participants indicated that they felt less stress or anxiety in such a homogeneous group as they did not need to cover or hide their skin there (e.g., A12, C14, E02, and K07). As E02 indicated:

I felt that everyone has something in common, so I don’t have to cover up my AD… We could feel happier when playing together. We are not so constrained. There is no need to hide. (E02, female, 10 years old, moderate)

Moreover, hope and expectations could be found in three participants’ drawings and verbal expressions in the second interviews (e.g., B06, K07, and G12). For example, poor school performance and interpersonal problems at school were the focus in the first interview with K07; however, she drew her “dream middle school” to express her hope for a new environment in the future in her second interview.

*Changes in physical experiences.* Many participants experienced high levels of disease severity with a severe and painful rash extensively affecting their body. Most of them complained about constant pruritus and sleep disturbance in the first interviews. Whereas seven participants directly indicated that their skin conditions were better than before in the second interviews (e.g., A12, C15, D06, D08, G16, H10, and K07). For example, K07 described the following: “It (AD) seems to be better after attending this group. I feel better. My AD is getting better”. As a result, three participants indicated that they could sleep better (e.g., C15, D06, and G16). In the first interview with D06, she drew and described her powerlessness and helplessness due to frequent sleep disturbances in her lived experiences. Living with severe AD meant extreme distress and horror for her. In the second interview, she was so excited to share that she could sleep well now. She described her second drawing:

This is what I am doing from 10:00pm to 7:00am. I won’t wake up… I used to wake up when I was itchy, but now I didn’t know as I was asleep. I am very happy… I have not slept well before, and I used to sleep very late. I was not happy. I was very sleepy when I was in class previously… I feel spirited in class now, so happy… I used to rub on the ointment before class, but now I don’t need to rub the ointment on at school…It is not that itchy. I am happy. My AD used to be very, very, very fierce… I thought AD in the past was a million times horrible. It is now ten times horrible. (D06, female, 8 years old, severe)

## 4. Discussion

Nonpharmaceutical interventions for children living with AD are very important due to the extensive and pervasive impact of the disease on their psychological and social well-being [2]. Using a drawing-based, qualitative approach, the current study examined the impact of a psychosocial intervention on the subjective experiences of 13 children diagnosed with moderate or severe AD in Hong Kong by describing the changes in their lived experiences before and after attending the intervention.

This study offered participant-driven insights into the effects of the IBMS intervention directly from the views of children living with AD. The views of children living with health problems have commonly been ignored in healthcare settings as they are usually seen as passive recipients [20]. It is very necessary to conduct qualitative studies to evaluate the effects of social services for any underrepresented group, such as young children living with AD in this study. Listening to children’s views has been perceived as an effective way for professionals to strengthen practices and move closer to the best services appropriate for children living with health problems [21,22]. This study highlighted the value of children’s perceptions in the evaluation of the professional services provided to them, and the strengths of drawing-based approaches for facilitating communication with children living with health problems in research as well.

**Figure 1:**
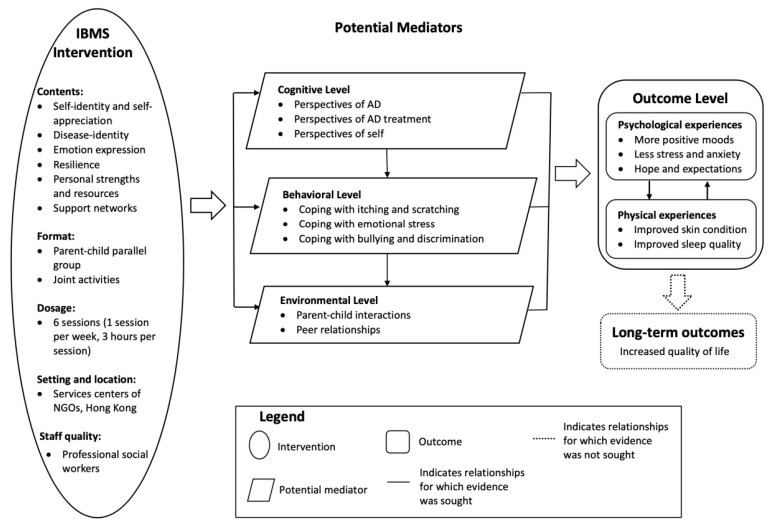
Potential mechanisms underlying the changes in participants’ subjective experiences.

By considering the connections and interactions among codes and themes, the findings served to generate hypotheses regarding the mechanisms of the changes in the subjective experiences of children living with AD. Figure 1 presents an explanation for how these mechanisms operated. The IBMS intervention affected participants as an integrated package, including its contents, format, dosage, setting, and staff quality. Of these components, its contents and format seemed to have overt effects while its dosage, setting, and staff quality might have covert effects.

In terms of the impact of its contents, changes occurred in participants’ perspectives on their disease, treatments, and themselves partially through the contents of the IBMS intervention. Children gained self-awareness from the self-identity session (session 1) of the intervention, which emphasized individual uniqueness, various personal strengths, and the sense of accomplishment. The philosophy throughout the disease identity session (session 2) of the IBMS intervention was to help participants face AD with a mindful attitude, cultivate equanimity in their disease journey, discover the meaning behind their sufferings, and embrace the vicissitudes in their lives, which also played an important role. Additionally, participants could also gain increased coping strategies with psychosocial difficulties and interpersonal difficulties partially from the contents of the IBMS intervention. Participants learned psychoeducational messages about body-mind connections, emotional regulation and simple mindfulness-based relaxation skills such as mindful breathing and mindful walking, and emotional management tools such as the mindfulness jar and emotional weather reports from the emotional expression session (session 3). By taking a precautionary approach, the IBMS intervention treated children as active participants with strengths. The resilience capacity session (session 4) and the personal strength session (session 5) of the IBMS intervention emphasized empowering participants’ internal capability and building resilience in adverse situations, especially when facing discrimination and school bullying. Moreover, the support networks session (session 6) played an important role in improving participants’ interpersonal relationships with peers and parents.

Besides the contents of the IBMS intervention, this study also highlighted the importance of the parallel group format of the intervention in achieving its effects. Social isolation and discrimination, which cause profound psychological harm, have been pervasive problems for children living with AD [23]. Besides the immediate pleasure and gratification gained from games and group activities designed according to their developmental stages, participants also acquired strategies for coping with itchiness and scratching from communication with peers with AD in the group. The group format built a mutual support network and created an accepting arena for these children [24]. Their new experiences of interacting with peers in the group activities further provoked their reinterpretation of the meaning of interpersonal relationships. The presence of AD in young children negatively affected the parent–child relationship and caused conflicts within the family [25]. The joint parent–child activities in each session strengthened the relationships and intensified mutual gratification between participants and their parents, thereby constructing an environment for unconditional love and trust in breeding positive emotions and habits of mutual appreciation. Secure parent–child relationships could provide a strong foundation for enhancing the effects of psychosocial interventions for children with chronic diseases [26].

The IBMS intervention worked by changing participants’ perceptions at the cognitive level, improving their coping strategies at the behavioral level, and constructing their social support network at the environmental level. Cognitive, behavioral, and environmental factors mediated the relationships between the IBMS intervention and participants’ psychological and physical outcomes. Participants’ positive self-identity combined with their mindful attitudes toward AD could not only reduce their anxiety about skin conditions but also encourage them to find meaning and benefit from AD treatments, leading to increased treatment compliance and decreased severity of AD. Participants applied their new coping strategies when handling physical difficulties such as scratching. Successful coping experiences could improve participants’ self-esteem, sense of control, and overall psychological well-being. The feeling of acting as a team with parents and new experiences in the homogeneous group not only played an important role in changing their understandings of AD but also brought positive emotions. They obviously felt happier and more satisfied with themselves in the new interpersonal context.

There was an interactive and reciprocal relationship between participants’ psychological well-being and their physical symptoms. The presence of AD could increase the possibility of psychological distress among children; furthermore, the presence of emotional stress could in turn induce or exacerbate AD, creating a vicious cycle [27,28]. The IBMS intervention treated the child with AD as a whole person and emphasized body–mind connections and emotional expressivity. Improved psychological well-being of participants in turn benefited their skin conditions and improved their sleep quality, creating a benign cycle. There would also seem to be a strong indication that working with the emotional dimensions should be a core component in the future design of nonpharmaceutical interventions for children living with AD.

### Limitations and Future Research

First, the small sample size might limit the generalizability of the findings. To minimize this limitation, we used maximum variation sampling in terms of participants’ age, gender, and severity of AD. Second, drawing was employed as a child-centered research method rather than a therapeutic tool in this study. However, the therapeutic impact of drawing on participants might exist due to children’s self-expression in the process, which might confound the effects of the IBMS intervention in this study. Third, although we believe that the IBMS services induced positive impact on the lived experiences of the participants, some other factors might be possible reasons for their changes. For example, the changes in B06 might be because he had just left his previous school and moved to a new school environment. H10 indicated that she felt less stressed because she had just finished her final examinations. Fourth, the current study did not describe the impact of participants’ age (younger vs. older) and disease severity (moderate vs. severe) on their subjective experiences and the effects of the IBMS intervention. Children’s chronological age and developmental stage affected not only their drawing skills but also their performances in interviews. The severity of AD was associated with children’s subjective experiences. Characteristics of participants should be considered more carefully in future research on the effects of psychosocial interventions.

## 5. Conclusions

With a drawing-based approach, this qualitative study contributed to an in-depth description of the impact of the IBMS intervention on the subjective experiences of children living with AD. This qualitative study highlighted the wider inclusion of child-centered qualitative research in the evaluation of the effects of psychosocial interventions designed for children. The current study also helped identify some active ingredients of the psychosocial intervention that should be implemented with fidelity when applying the findings to other settings.

## Figures and Tables

**Table 1:** Process of sampling.

Variables			Service Centers								Selected Participants
Age (Year)	Gender	Severity of AD	A	B	C	D	E	G	H	K	
8	Male	moderate				D02					D02
9	Male	moderate				D08			H12	K18	D08
10	Male	moderate									/
11	Male	moderate								K12	K12
12	Male	moderate		B06							B06
8	Male	severe	A07				E09	G16	H22		G16
9	Male	severe							H14	K04	H14 → H09
10	Male	severe	A11		C15			G04	H09		C15
11	Male	severe						G12			G12
12	Male	severe		B03, B05	C16						B05
8	Female	moderate									/
9	Female	moderate				D04			H08		H08
10	Female	moderate					E02				E02
11	Female	moderate									/
12	Female	moderate									/
8	Female	severe				D06					D06
9	Female	severe	A12		C07, C13	D14			H20		H20 + A12
10	Female	severe			C08				H10, H11		H10
11	Female	severe								K07, K13	K07
12	Female	severe			C14				H07		C14

**Table 2:** Characteristics of participating children.

ID	Gender	Age (Year)	Total SCORAD Score	Severity of AD	Age at Diagnosis (Month)	Treatments Received	Other Conditions	Monthly Family Income (HKD)	NO. of Children at Home	NO. of Children with AD	Interview Duration (min)	
											Time I	Time II
A12	Female	9	78	Severe	6	123456	/	NR	2	2	75	52
B06	Male	12	43	Moderate	20	123467	/	20,000–29,999	3	1	34	50
C14	Female	12	94	Severe	3	12356	/	30,000–39,999	2	2	57	88
C15	Male	10	74	Severe	84	126	ADHD	NR	2	2	67	71
D06	Female	8	71	Severe	1	12356	/	60,000–69,999	2	1	65	68
D08	Male	9	46	Moderate	12	12367	ASD	30,000–39,999	2	1	23	29
E02	Female	10	40	Moderate	0	123456	/	>or =80,000	2	2	62	60
G12	Male	11	78	Severe	2	1234567	/	10,000–19,999	1	1	66	48
G16	Male	8	63	Severe	3	1246	Asthma	20,000–29,999	2	2	47	62
H08	Female	9	25	Moderate	36	123456	/	>or =80,000	1	1	66	46
H10	Female	10	63	Severe	48	1236	AR	NR	4	3	22	40
H20	Female	9	77	Severe	1	1236	/	>or =80,000	1	1	60	41
K07	Female	11	82	Severe	3	123456	/	20,000–29,999	2	1	54	31

Note: Treatments received: 1 = ointment, 2 = partial medication (steroid), 3 = partial medication (non-steroid), 4 = wet wrap therapy, 5 = oral antibiotic, 6 = traditional Chinese medicine (TCM), 7 = others; Other conditions: ADHD = attention-deficit/hyperactivity disorder; ASD = autism spectrum disorder; AR = allergic rhinitis.

**Table 3:** A codebook.

Themes and Codes
Theme 1: Physical experiences
1. Itching and scratching
2. Sleep disturbance
3. Visible skin symptoms and disfigurement
4. Chronic and relapsing nature
5. Pain
6. Short stature
7. Daily life challenges
Theme 2: Psychological experiences
1. Angry or annoyed
2. Sad or unhappy
3. Worried or afraid
4. Stressed
5. Embarrassed
6. Confused
Theme 3: Cognitive experiences
1. Perceptions of living with AD
2. Perceptions of AD treatment
3. Perceptions of self
4. Perceived interpersonal relationships
Theme 4: Social experiences
1. Relationships with parents
2. Relationships with siblings
3. Relationships with peers
4. Relationship with teachers
5. Relationship with others
Theme 5: Coping strategies
1. Coping with itching and scratching
2. Coping with emotional stress
3. Coping with discrimination or bullying
Theme 6: Experiences in IBMS
1. Games and group activities
2. Parent–child joint activities

## Data Availability

The datasets generated during and/or analyzed during the current study are not publicly available due to individual privacy but are available from the corresponding author on reasonable request.
